# Association between Neurofibromatosis Type 1 and Breast Cancer: A Report of Two Cases with a Review of the Literature

**DOI:** 10.1155/2015/456205

**Published:** 2015-10-29

**Authors:** Yoon Nae Seo, Young Mi Park

**Affiliations:** Department of Radiology, Inje University Busan Paik Hospital, 633-165 Gaegeum-dong, Busanjin-gu, Busan 614-735, Republic of Korea

## Abstract

Neurofibromatosis type 1 (NF1) is one of the most common genetic diseases in humans and is associated with various benign and malignant tumors, including breast cancer. However, an increased risk of breast cancer in NF1 patients has not been widely recognized or accepted. Here, we report two cases of breast cancer in NF1 patients and review the literature on the association between NF1 and breast cancer.

## 1. Introduction

Neurofibromatosis type 1 (NF1) or von Recklinghausen disease is one of the most common autosomal dominant diseases in humans, and its incidence and prevalence have been reported to be approximately 1 in 2,700 and 1 in 4,600, respectively [1]. NF1 is a group of heterogeneous multisystem neurocutaneous disorders and is caused by mutations in the* NF1* gene, which is considered a classical tumor suppressor. Besides the development of neurofibromas, which are benign peripheral nerve sheath tumors, NF1 patients have an increased risk of developing other benign and malignant neoplasms. Breast cancer has been shown to be associated with NF1 [2–7]; however, an increased risk of breast cancer in NF1 patients has not been widely recognized or accepted.

Here, we report two cases of breast cancer in NF1 patients and review the literature on the association between NF1 and breast cancer.

## 2. Case Presentation

### 2.1. Case One

A 25-year-old woman with a palpable lump in her right breast was referred to our department. She had been diagnosed with NF1 at the age of 17 years. Two of her aunts had cancer; one had breast cancer and the other had ovarian cancer. However, there was no family history of neurofibromatosis.

**Figure 1:**
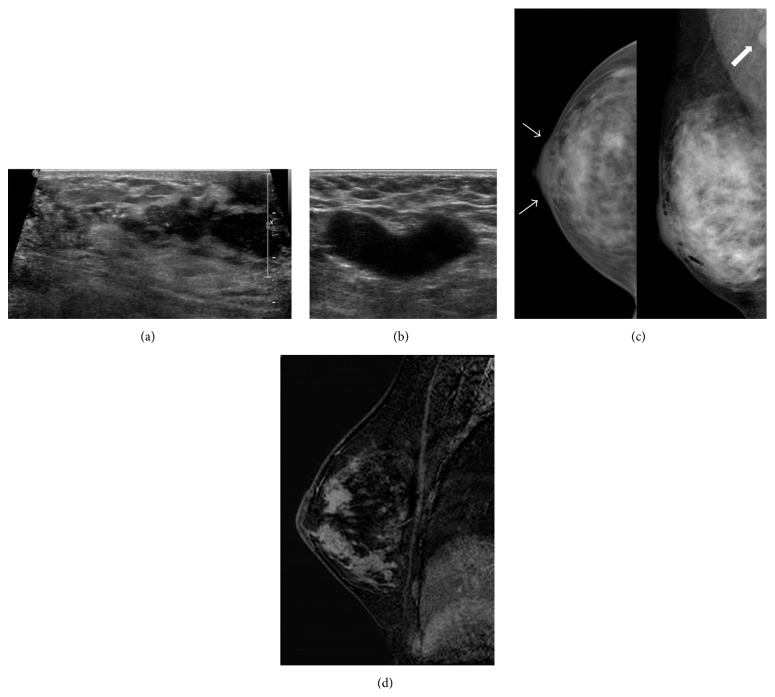
Ultrasonography shows an ill-defined hypoechoic mass with microcalcifications and irregular duct changes in the right breast. The mass with irregular duct changes extends to the subareolar area (a). Metastatic lymph node is seen in the ipsilateral axilla (b). Mammography shows fine pleomorphic microcalcifications with segmental distribution in the lower outer portion of the right breast. And mild skin thickening (arrows in c) and metastatic lymph node are also noted (c). Magnetic resonance imaging shows that lesion exhibits nonmass enhancement lesion with heterogeneous internal enhancement pattern and occupies most of the right breast, except the lower inner portion (d).

On US, an ill-defined hypoechoic mass with microcalcifications and irregular duct changes, extending to the subareolar area, was noted in her right breast. Additionally, several lesions believed to be metastatic lymph nodes were observed in the ipsilateral axilla (Figures 1(a) and 1(b)). On mammography (MMG), fine pleomorphic microcalcifications with segmental distribution were noted in the lower outer portion of her right breast (Figure 1(c)). On MRI, the lesion showed about 6.5 cm sized, nonmass enhancement lesion with heterogeneous internal enhancement pattern and occupied most of the right breast, except the lower inner portion (Figure 1(d)).

She underwent US-guided core needle biopsy in the lower outer portion of her right breast and of the pathological lymph nodes in the right axilla. She was diagnosed with ductal carcinoma in situ in the breast and metastatic lymphadenopathy in the right axilla. She underwent modified radical mastectomy and axillary lymph node dissection, and the final diagnosis was invasive ductal carcinoma with axillary metastasis (T2N3M0; estrogen receptor positive; progesterone receptor positive; human epidermal growth factor receptor 2 negative; Ki-67 10–20%). We analyzed DNA from peripheral blood in order to evaluate the presence of mutations in the* BRCA1* and* BRCA2* genes. Specific coding regions and exon-intron boundaries of the* BRCA1* and* BRCA2* genes were amplified using polymerase chain reaction (PCR). Sequence alterations were confirmed at the genomic level with PCR amplification, and no mutation was noted in the* BRCA1* or* BRCA2* gene. She received postoperative chemotherapy and radiation therapy. Presently, she is being regularly followed up, and she has not shown any signs of disease recurrence.

### 2.2. Case Two

**Figure 2:**
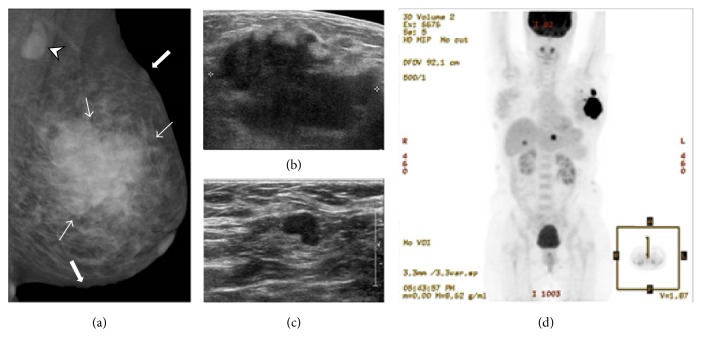
Left mediolateral oblique mammography shows an irregular hyperdense mass with indistinct margin (thin arrow) and axillary lymphadenopathy (arrowhead). Additionally, skin undulation is seen, indicating presence of cutaneous neurofibromas (thick arrow). Ultrasonography shows a large, hypoechoic, irregular mass with angular margin in the left breast (b) and multiple metastatic lymph nodes in the left axilla (c). PET-CT shows hepatic metastasis (d).

A 47-year-old woman with NF1 visited our department for a palpable lump in her left breast. On MMG, an irregular hyperdense mass with microlobulated margin was noted in her left breast, and pathologic lymph nodes were noted in her left axilla (Figure 2(a)). On US, the large mass appeared to be of irregular shape, angular margin, and hypoechoic echotexture and multiple pathological lymph nodes were noted in the left axilla at levels I and II (Figures 2(b) and 2(c)).

She underwent US-guided core needle biopsy of the irregular mass in the left breast and was diagnosed with invasive ductal carcinoma. Additionally, during the staging workup, she was diagnosed with hepatic metastasis on PET-CT (Figure 2(d)). She received neoadjuvant chemotherapy and later underwent modified radical mastectomy and axillary lymph node dissection. The final diagnosis was invasive ductal carcinoma with axillary lymph node metastasis (T3N1M1; estrogen receptor negative; progesterone receptor negative; human epidermal growth factor receptor 2 negative; Ki-67 10–20%). During follow-up, the hepatic metastasis worsened and lung, bone, and retroperitoneal lymph node metastases were diagnosed. She eventually died 15 months after being diagnosed with breast cancer.

## 3. Discussion

Here, we reported two cases of NF1 associated with breast cancer. NF1 is a complex neuroectodermal disorder characterized by autosomal dominant inheritance, high penetrance, and wide variability in expression. The disease is caused by mutations in the* NF1* gene, and the risk of various types of tumors, especially those derived from the embryonic neural crest, including pheochromocytoma, leukemia, glioma, rhabdomyosarcoma, astrocytoma, and malignant peripheral nerve sheath tumor, is higher in individuals with* NF1* mutations than in the general population [5].

The* NF1* gene is located in the pericentromeric region of the long arm of chromosome 17, which interestingly also includes the* BRCA1* gene. An interaction between these two genes has been suggested [4]; however, the exact interaction is unclear.

Neurofibromin, the protein product of the* NF1* gene, functions as a negative regulator of the Ras oncogene pathway, interacting with Ras and converting active Ras-GTP to its inactive form Ras-GDP. Ras is an essential component of the signal transduction pathways that regulate cell growth, proliferation, differentiation, and apoptosis, and the impairment of the hydrolytic reaction is associated with an increased risk of cancer. Hence, the* NF1* gene has a potential role as a tumor suppressor gene [29, 39].

The first report of an association between NF1 and breast cancer was published in 1972 [8], and subsequently several clinical cases of NF1 patients with breast cancer have been reported in the literature. We reviewed the English literature and have summarized all the reports of breast cancer in NF1 patients in Table 1 [3–38]. There were 29 cases of breast cancer in NF1 patients and six studies about the association between breast cancer and NF1. Among 20 patients whose age at diagnosis was reported, eight patients (40%) were diagnosed with breast cancer before 40 years of age and five patients (25%) were diagnosed before 30 years of age. Additionally, among 19 patients whose breast cancer stage was reported, 10 patients (52.6%) had advanced cancer (greater than stage IIB). In the six studies, the incidence of breast cancer was reported to be 1.1–19.7% [2–7].

Sharif et al. retrospectively evaluated the risk of developing breast cancer among 304 women aged 20 years or older who were diagnosed with NF1 over a period of 30 years [4]. These authors found that the overall standardized incidence ratio of breast cancer was 3.5 in women with NF1 and that the risk of developing breast cancer at the age of 50 years was 4.9-fold higher in women with NF1 than in women in the general population. Additionally, the cumulative risk of developing breast cancer at the age of 50 years was 2% in women in the general population and was 8.4% in women with NF1 [4]. Similarly, Madanikia et al. retrospectively evaluated the risk of developing breast cancer among 126 women aged 20 years or older who were diagnosed with NF1 over a period of 15 years [5]. These authors found that the overall incidence of breast cancer in women with NF1 was 3.2%. Additionally, the risk of breast cancer was nearly threefold higher in women with NF1 who were under 50 years old than in women in the general population [5]. Nakamura et al. noted that breast cancer occurred in 18.5% of young women (<35 years of age) with NF1, which is a relatively high incidence when compared to the incidence of 6.7% in young women (<35 years of age) without NF1 reported in another study [14]. Both of our patients developed breast cancer under 50 years of age, and one of these patients developed breast cancer at 25 years of age. A previous article reported the development of breast cancer in a 21-year-old woman with* NF1* and* BRCA1* mutations [37]. However, our patients did not have* BRCA* gene mutations.

Murayama et al. reported 37 cases of breast cancer associated with NF1, and most of the cases were diagnosed at an advanced stage [15]. In both of our patients, breast cancer was diagnosed at an advanced stage (stage IIIC in one case and stage IV in the other case). Furthermore, Evans et al. reported that women with NF1 have not only an increased risk of breast cancer but also an increased rate of mortality associated with breast cancer diagnosis [40].

All the above-mentioned articles have similar findings that NF1 increases the risk of developing breast cancer and that NF1 patients with breast cancer have a poor prognosis.

Breast cancer screening guidelines have been well established for the general population and for high-risk women, to decrease mortality through early diagnosis. However, currently, there are no such guidelines for NF1 patients, and guidelines similar to those for Cowden syndrome, which is a genetic disorder associated with breast cancer, should be developed [41].

## 4. Conclusion

The patients and physicians should be aware of the high possibility of breast cancer in individuals with NF1. For early diagnosis, the current guidelines used to screen women in the general population appear to be insufficient to screen NF1 patients. The findings of the above-mentioned reports and other published data justify the requirement of specific screening programs for NF1 patients, similar to the programs for Cowden syndrome patients. Further studies are needed to clarify the relationship between NF1 and breast cancer, especially at the genetic level, and to establish specific screening guidelines for the early diagnosis of breast cancer in NF1 patients.

## Figures and Tables

**Table 1:** Summary of previously reported NF1 patients with breast cancer.

Author	Age (yr)	Family history of breast cancer	Gene mutation	Stage	Molecular subtype	Characteristics
Brasfield and Das Gupta [8]	5 patients					
McMillan and Edwards [9]	27	NA	NA	NA	NA	
Hiraide et al. [10]	32	NA	NA	NA	NA	
el-Zawahry et al. [11]	2 patients					
Vilar Sanchis and Vazquez Albaladejo [12]	1 patient					
Hollo way et al. [13]	68	NA	NA	IIA	NA	Male
Nakamura et al. [14]	49	NA	NA	NA	NA	
Murayama et al. [15]	66	NA	NA	IIA	NA	
Ceccaroni et al. [16]	66	Daughter^*∗*^	NA	NA	NA	
Satgé et al. [17]	23	4 aunts	NA	NA	NA	
Güran and Safali [18]	23^a^	Mother^*∗*^	NA	NA	NA	
	52^a^	Daughter^*∗*^	NA	NA	NA	
Posada and Chakmakjian [19]	74	No	NA	IIA	NA	
Kawawa et al. [20]	66	No	NA	IIB	Luminal	Paget' disease
Natsiopoulos et al. [21]	60	No	NA	IIB		Metaplastic carcinoma
Hasson et al. [22]	49	No	NA	IB	Luminal	
Invernizzi et al. [23]	60	NA	NA	IA	Luminal	
Alamsamimi et al. [24]	51	Sister	NA	IIA	Luminal	Synchronous bilateral breast cancer
Salemis et al. [25]	59	No	NA	IIB	Luminal	
Bhargava et al. [26]	58	NA	NA	NA	NA	
Takeuchi et al. [27]	76	NA	NA	IIA	NA	Metachronous contralateral breast cancer
Zhou et al. [28]	48	NA	NA	IA	Luminal	
Campos et al. [29]	35^a^	Mother	NA	NA	Nonluminal	
	40^a^	Daughter	BRCA1	IV	NA	
Cheuk et al. [30]	42	NA	NA	NA	NA	
Jinkala et al. [31]	69	NA	NA	NA	NA	
Nogimori et al. [32]	1 patient					
Vivas et al. [33]	53	NA	NA	IV	HER2	Metaplastic carcinoma
Lakshmaiah et al. [34]	55	No	NA	IIB	Luminal	Male
Chaudhry et al. [35]	46	No	NA	IIIA	HER2	Metaplastic carcinoma
Da Silva et al. [36]	54	No	NA	IA	HER2	
Jeon et al. [37]	21^a^	No	No	IIB	Luminal	
	30^a^	No	NA	IIA	Luminal	Metachronous contralateral breast cancer
Khalil et al. [38]	39	NA	NA	IIIA	Luminal	
Zöller et al. [2]	2 breast cancers in 70 NF1 patients (2.8%)					
Walker et al. [3]	5 breast cancers in 448 NF1 patients (1.1%)					
Sharif et al. [4]	14 breast cancers in 304 NF1 patients (4.6%)					
Madanikia et al. [5]	4 breast cancers in 126 NF1 patients (3.2%)					
Wang et al. [6]	15 breast cancers in 76 NF1 patients (19.7%)					
Seminog and Goldacre [7]	52 breast cancers in 3855 NF1 patients (1.3%)					

NF1: neurofibromatosis type 1; NA: not assessable; luminal: estrogen receptor (ER) or progesterone receptor (PR) positive; nonluminal: ER and PR negative; HER2: ER and PR negative and human epidermal growth factor receptor 2 (HER2) positive.

^*∗*^Neurofibromatosis patient.

^a^Mother-daughter relationship.
